# Smoking, diet, pregnancy and oral contraceptive use as risk factors for cervical intra-epithelial neoplasia in relation to human papillomavirus infection

**DOI:** 10.1054/bjoc.1999.1100

**Published:** 2000-03-06

**Authors:** L Kjellberg, G Hallmans, A-M Åhren, R Johansson, F Bergman, G Wadell, T Ångström, J Dillner

**Affiliations:** Departments of Obstetrics and Gynecology, Public Health and Clinical Medicine, Pathology, Clinical Virology, Clinical Cytology, University Hospital of Northern Sweden, Umeå, S-901 85, Sweden; The Microbiology and Tumor Biology Center, Karolinska Institute, Stockholm, Sweden

**Keywords:** risk factors, cervical intraepithelial neoplasia, human papillomavirus infection

## Abstract

Smoking, nutrition, parity and oral contraceptive use have been reported as major environmental risk factors for cervical cancer. After the discovery of the very strong link between human papillomavirus (HPV) infection and cervical cancer, it is unclear whether the association of these environmental factors with cervical cancer reflect secondary associations attributable to confounding by HPV, if they are independent risk factors or whether they may act as cofactors to HPV infection in cervical carcinogenesis. To investigate this issue, we performed a population-based case–control study in the Västerbotten county of Northern Sweden of 137 women with high-grade cervical intra-epithelial neoplasia (CIN 2–3) and 253 healthy age-matched women. The women answered a 94-item questionnaire on diet, smoking, oral contraceptive use and sexual history and donated specimens for diagnosis of present HPV infection (nested polymerase chain reaction on cervical brush samples) and for past or present HPV infections (HPV seropositivity). The previously described protective effects of dietary micronutrients were not detected. Pregnancy appeared to be a risk factor in the multivariate analysis (*P*< 0.0001). Prolonged oral contraceptive use and sexual history were associated with CIN 2–3 in univariate analysis, but these associations lost significance after taking HPV into account. Smoking was associated with CIN 2–3 (odds ratio (OR) 2.6, 95% confidence interval (CI) 1.7–4.0), the effect was dose-dependent (*P* = 0.002) and the smoking-associated risk was not affected by adjusting for HPV, neither when adjusting for HPV DNA (OR 2.5, CI 1.3–4.9) nor when adjusting for HPV seropositivity (OR 3.0, CI 1.9–4.7). In conclusion, after taking HPV into account, smoking appeared to be the most significant environmental risk factor for cervical neoplasia. © 2000 Cancer Research Campaign


*British Journal of Cancer* (2000) **82**(7), 1332–1338
© 2000 Cancer Research Campaign
DOI: 10.1054/ bjoc.1999.1100, available online at http://www.idealibrary.com on IDEAL®

### Smoking, diet, pregnancy and oral contraceptive use as risk factors for cervical intra-epithelial neoplasia in relation to human papillomavirus infection

**L Kjellberg[1], G Hallmans[2], A-M Åhren[2], R Johansson[2], F Bergman[3], G Wadell[3], T Ångström[5] and J Dillner[2,6]**

Departments of [1]Obstetrics and Gynecology, [2]Public Health and Clinical Medicine, [3]Pathology, [4]Clinical Virology and [5]Clinical Cytology, University Hospital of Northern Sweden, S-901 85 Umeå, Sweden; [6]The Microbiology and Tumor Biology Center, Karolinska Institute, Stockholm, Sweden

**Summary** Smoking, nutrition, parity and oral contraceptive use have been reported as major environmental risk factors for cervical cancer. After the discovery of the very strong link between human papillomavirus (HPV) infection and cervical cancer, it is unclear whether the association of these environmental factors with cervical cancer reflect secondary associations attributable to confounding by HPV, if they are independent risk factors or whether they may act as cofactors to HPV infection in cervical carcinogenesis. To investigate this issue, we performed a population-based case–control study in the Västerbotten county of Northern Sweden of 137 women with high-grade cervical intra-epithelial neoplasia (CIN 2–3) and 253 healthy age-matched women. The women answered a 94-item questionnaire on diet, smoking, oral contraceptive use and sexual history and donated specimens for diagnosis of present HPV infection (nested polymerase chain reaction on cervical brush samples) and for past or present HPV infections (HPV seropositivity). The previously described protective effects of dietary micronutrients were not detected. Pregnancy appeared to be a risk factor in the multivariate analysis ($P < 0.0001$). Prolonged oral contraceptive use and sexual history were associated with CIN 2–3 in univariate analysis, but these associations lost significance after taking HPV into account. Smoking was associated with CIN 2–3 (odds ratio (OR) 2.6, 95% confidence interval (CI) 1.7–4.0), the effect was dose-dependent ($P = 0.002$) and the smoking-associated risk was not affected by adjusting for HPV, neither when adjusting for HPV DNA (OR 2.5, CI 1.3–4.9) nor when adjusting for HPV seropositivity (OR 3.0, CI 1.9–4.7). In conclusion, after taking HPV into account, smoking appeared to be the most significant environmental risk factor for cervical neoplasia. © 2000 Cancer Research Campaign

**Keywords:** risk factors; cervical intraepithelial neoplasia; human papillomavirus infection

Infection with the oncogenic types of human papillomavirus (HPV) has been established as a main cause of high-grade cervical intra-epithelial neoplasia (CIN 2–3), an immediate precursor of cervical cancer (Lorincz et al, 1992; Cuzick et al, 1994). The time lag between infection and development of invasive cervical cancer is probably on average more than 15 years (Östör, 1993). Most HPV infections clear spontaneously but a minority of infections, especially of high-risk types, will persist and those are a major risk factor for development of CIN 2–3 (Ho et al, 1998). The risk factors that determine whether HPV infection will be transient or become persistent as well as the risk factors that determine progression from CIN to invasive cervical cancer are insufficiently known.

Since certain risk factors for cervical cancer or CIN such as smoking and oral contraceptive use have been reported to be associated with HPV infection (Hildesheim et al, 1990; Butterworth et al, 1992; Burger et al, 1993; Eluf-Neto et al, 1994; Ho et al, 1998; Kruger-Kjaer et al, 1998; Kwasniewska et al, 1998; Olsen et al, 1998) this may have confounded the risk ratios. The transient nature of HPV infections makes epidemiological studies of HPV exposure as such difficult, since women currently negative for HPV DNA may have been infected previously. However, the serum antibody response to HPV capsids will persist on long-term follow-up, even after HPV infection is no longer found (af Geijersstam et al, 1998) and is a useful marker of life-time HPV exposure (Olsen et al, 1997). As most previous studies have either not taken HPV into account or only studied present infection, we conducted a population-based case–control study of CIN 2–3 in Northern Sweden, taking past and present HPV exposures into account.

*Received 22 June 1999*
*Revised 4 October 1999*
*Accepted 26 October 1999*

*Correspondence to:* L Kjellberg

### SUBJECTS AND METHODS

### Study base

The Västerbotten county in Northern Sweden has 257 079 inhabitants (1993). The population-based cervical screening programme started in 1969 and all women resident in the county aged 25–59 years are invited by letter for screening every three years. The participation rate is about 80%. The mean number of women living in this area and aged between 25 and 59 years of age was about 57 000.

### Screening procedure

Cytologic samples, taken at regional health care centres by midwives, were examined in the laboratory of Clinical Cytology, Umeå University Hospital, the classification criteria being those formulated by Koss (1979).

### Study groups

*Referral group*

Between October 1993 and December 1995, 254 women with a pathological cervical smear, referred for colposcopy in Västerbotten county were asked to participate. One hundred and seventy-four women were referred from the population-based cervical screening programme and 76 because of smears taken outside the programme. They were referred to any of the three hospitals in Västerbotten county: Umeå University Hospital, Skellefteå Community Hospital or Lycksele Community Hospital. Four of the referred women refused to participate in the study, and the remaining 250 were enrolled. One hundred and thirty-seven of these women were finally diagnosed with CIN 2–3. The final diagnosis was based on blinded histopathological re-classification by an experienced pathologist of stored archival biopsy specimens (130 cases) and for seven cases where an archival biopsy was not available, on a blinded reclassification of the cytological archival specimen. The mean age was 37.2 years (range 21–58).

*Screening group*

A group of women who were invited by letter for screening in the organized programme were asked by an additional enclosed letter to attend the study. The women were selected randomly from the population registry and matched to the referrals 1:1 for age (±3 years) and area of residence. From October 1995 until March 1996, out of 2191 invited women, 871 were asked to participate and 320 accepted. In order to find out the reason 63% did not accept the invitation, a randomly selected subgroup of 50 of these women was interviewed by telephone by the same investigator. Two were not interested to participate in the screening programme at all. The other 48 women had recently seen or intended to see a doctor or midwife for screening and did not want to participate because of inconvenience.

### Sampling procedures

In January 1995, the organized screening programme switched cervical sampling device to Cervex brush (Cortec Medical AB, Malmö, Sweden) that permits collecting cell samples from both ecto- and endocervix. The 114 samples that were taken before that time were taken with a cotton-tipped pin in the endocervix and a wooden spatula from the ectocervix and posterior fornix. Before colposcopic examination, a sample for HPV DNA was taken from the endocervix by a rotary motion with a Cytobrush (Medscand, Malmö, Sweden) for detection of cervical HPV DNA by nested polymerase chain reaction (PCR) with consensus primers and type-specific primers for HPV 11, 16, 18 and 33. The HPV sample was stored in physiologic saline containing 10 mM Tris–HCl buffer, pH 7.8 and kept at –70°C until analysis. If the colposcopic examination was normal an annual follow-up was arranged.

In the screening population, all women were examined by the same physician. Samples for cytomorphology and HPV DNA detection was taken and stored in the same manner as for referrals after January 1995. Although the sample for HPV DNA detection was taken identically for all cases and controls, the fact that the 114 cases sampled in 1993–1994 had had their previous Pap smear taken with a different utensil might have introduced differential misclassification bias. This was investigated by comparing the HPV results for cases sampled before and after January 1995, and no differences were found (Kjellberg et al, 1998).

### Questionnaire

The women answered a questionnaire concerning dietary habits, tobacco use, health conditions, psychosocial situation and an 11-item questionnaire about sexual and reproductive history.

The food frequency part included 84 items with an increasing nine-level scale, ranging from never eaten to eaten four or more times per day and the respondents were asked to report their usual intake of food during the last year. A standardized portion size adjusted for sex and age was used.

The energy and nutrients contents were calculated using the database from the National Food Administration (Bergström et al, 1991). The reported frequencies of consumption were converted into number of intakes per day and the total intake of energy and nutrients was estimated.

Missing answers in the food groups with several alternative items and where one of the alternatives was eaten frequently, such as milk products and types of fats for bread, were interpreted as being never eaten.

The food frequency questionnaire has been extensively validated previously (Johansson et al, 1994; submitted).

### Analysis

HPV PCR analysis, HPV typing and antibody analysis were carried out as described previously (Kjellberg et al, 1998).

### Statistical analysis

Using the Statistical Package for the Social Sciences (SPSS) program software, conditional logistic regression analysis was used to calculate the odds ratios (OR) with 95% confidence interval (CI).

### RESULTS

A total of 543 women answered the food questionnaire and 448 were eligible as cases (histological diagnosis of CIN 2–3) or controls (normal cytology). Nineteen women had too many missed answers (> 6 items) and five reported an unrealistic energy intake (<500 kcal or > 3500 kcal) and were therefore excluded. Five hundred and forty women had answered the questionnaire about smoking habits and 468 were eligible as cases or controls. The questionnaire about sexual history was answered by 556 women of which 480 were eligible. The distribution of dietary intake among women with normal cervical cytology and with CIN 2–3 is presented in Table 1. Drinking boiled coffee and a high intake of dairy products were the only significant positive associations. The point estimates changed only marginally after taking HPV and smoking into account (data not shown). A reduced risk associated with tea drinking and increased intake of vegetable fibres was not significant after adjusting for HPV DNA and smoking (Table 1).

A prolonged period of oral contraceptive (OC) use (more than 5 years) was associated with an increased risk for CIN 2–3 ($P < 0.05$), but this risk disappeared after adjustment for HPV, smoking and age (Table 2). Since OC use has been postulated to act via influencing the folate status (Butterworth et al, 1982; Harper et al, 1994) the possibility of an interaction between OC use and folate status was investigated. There was no OC-associated risk for CIN 2–3, neither among women with high, nor among women with low, folate intake (data not shown).

© 2000 Cancer Research Campaign

**Table 1** Odds ratios of CIN 2–3 according to dietary intake of fat and fibre from various sources and selected vitamins

| | Normal | CIN 2–3 | Total number | OR | Adj OR[a] | Adj OR[b] |
|---|---|---|---|---|---|---|
| Fat total (quartiles) | | | | | | |
| g day$^{-1}$ < 43.7 | 60 (75%) | 20 (25%) | 80 | 1.0 (*n* = 321) | 1.0 (*n* = 316) | 1.0 (*n* = 311) |
| 43.7 ≤ 55.5 g day$^{-1}$ | 60 (75%) | 20 (25%) | 80 | 1.0 (0.5–2.0) | 0.6 (0.2–2.2) | 1.0 (0.4–2.1) |
| 55.5 ≤ 70.9 g day$^{-1}$ | 61 (75%) | 20 (25%) | 81 | 1.0 (0.5–2.0) | 0.3 (0.1–1.2) | 0.8 (0.3–1.7) |
| day$^{-1}$ ≥ 70.9 g | 53 (66%) | 27 (34%) | 80 | 1.5 (0.8–3.0) | 0.9 (0.3–3.2) | 1.5 (0.7–3.5) |
| Missing | 98 | 29 | 127 | | | |
| *P* for trend | | | | 0.24 | 0.71 | 0.45 |
| Fat, saturated (quartiles) | | | | | | |
| g day$^{-1}$ < 18.5 | 60 (72%) | 23 (28%) | 83 | 1.0 (*n* = 332) | 1.0 (*n* = 326) | 1.0 (*n* = 321) |
| 18.5 ≤ 24.3 g day$^{-1}$ | 62 (75%) | 21 (25%) | 83 | 0.9 (0.4–1.8) | 1.0 (0.3–3.2) | 0.7 (0.3–1.6) |
| 24.3 ≤ 31.2 g day$^{-1}$ | 68 (81%) | 16 (19%) | 84 | 0.6 (0.3–1.3) | 0.7 (0.2–2.1) | 0.5 (0.2–1.2) |
| day$^{-1}$ ≥ 31.2 g | 53 (65%) | 29 (35%) | 82 | 1.4 (0.7–2.8) | 1.2 (0.4–3.7) | 1.3 (0.6–2.9) |
| Missing | 89 | 27 | 116 | | | |
| *P* for trend | | | | 0.45 | 0.94 | 0.65 |
| Fat, monounsaturated (quartiles) | | | | | | |
| g day$^{-1}$ < 15.5 | 63 (76%) | 20 (24%) | 83 | 1.0 (*n* = 333) | 1.0 (*n* = 327) | 1.0 (*n* = 322) |
| 15.5 ≤ 19.6 g day$^{-1}$ | 61 (75%) | 20 (25%) | 81 | 1.0 (0.5–2.1) | 0.8 (0.3–2.6) | 1.0 (0.4–2.2) |
| 19.6 ≤ 24.7 g day$^{-1}$ | 63 (74%) | 22 (26%) | 85 | 1.1 (0.5–2.2) | 0.7 (0.2–2.2) | 1.0 (0.4–2.1) |
| g day$^{-1}$≥ 24.7 | 57 (68%) | 27 (32%) | 84 | 1.5 (0.8–2.9) | 0.9 (0.3–2.9) | 1.4 (0.6–3.2) |
| Missing | 88 | 27 | 115 | | | |
| *P* for trend | | | | 0.24 | 0.85 | 0.42 |
| Fat, polyunsaturated (quartiles) | | | | | | |
| g day$^{-1}$ < 5.93 | 58 (71%) | 24 (29%) | 82 | 1.0 (*n* = 329) | 1.0 (*n* = 324) | 1.0 (*n* = 319) |
| 5.93 ≤ 7.67 g day$^{-1}$ | 66 (81%) | 16 (19% | 82 | 0.6 (0.3–1.2) | 0.3 (0.1–1.1) | 0.5 (0.2–1.1) |
| 7.67 ≤$^{1}$ 9.74 g day$^{-}$ | 57 (69%) | 26 (31%) | 83 | 1.1 (0.6–2.1) | 0.9 (0.3–2.7) | 0.8 (0.4–1.7) |
| day$^{-1}$ ≥ 9.74 g | 58 (71%) | 24 (29%) | 82 | 1.0 (0.5–2.0) | 0.9 (0.3–2.7) | 1.0 (0.4–2.1) |
| Missing | 93 | 26 | 119 | | | |
| *P* for trend | | | | 0.59 | 0.90 | 0.82 |
| Fibre, total (quartiles) | | | | | | |
| g day$^{-1}$ < 11.55 | 65 (71%) | 26 (29%) | 91 | 1.0 (*n* = 366) | 1.0 (*n* = 361) | 1.0 (*n* = 356) |
| 11.55 ≤ 14.63 g day$^{-1}$ | 66 (72%) | 26 (28%) | 92 | 1.0 (0.5–1.9) | 0.5 (0.1–1.5) | 1.2 (0.6–2.5) |
| 14.63 ≤ 19.13 g day$^{-1}$ | 69 (75%) | 23 (25%) | 92 | 0.8 (0.4–1.6) | 0.4 (0.1–1.3) | 1.1 (0.5–2.2) |
| g day$^{-1}$≥ 19.13 | 71 (78%) | 20 (22%) | 91 | 0.7 (0.4–1.4) | 1.6 (0.5–5.2) | 1.2 (0.6–2.6) |
| Missing | 61 | 21 | 82 | | | |
| *P* for trend | | | | 0.26 | 0.58 | 0.75 |
| Cereal fibre (quartiles) | | | | | | |
| g day$^{-1}$ < 4.23 | 79 (77%) | 24 (23%) | 103 | 1.0 (*n* = 412) | 1.0 (*n* = 405) | 1.0 (*n* = 401) |
| 4.23 ≤ 6.72 g day$^{-1}$ | 69 (67%) | 34 (33%) | 103 | 1.6 (0.9–3.0) | 0.6 (0.2–1.7) | 1.7 (0.9–3.3) |
| 6.72 ≤ 9.49 g day$^{-1}$ | 75 (73%) | 28 (27%) | 103 | 1.2 (0.6–2.3) | 0.6 (0.2–1.7) | 1.3 (0.7–2.7) |
| g day$^{-1}$ ≥ 9.49 | 84 (82%) | 19 (18%) | 103 | 0.7 (0.4–1.5) | 0.9 (0.3–2.5) | 1.0 (0.5–2.2) |
| Missing | 25 | 11 | 36 | | | |
| *P* for trend | | | | 0.29 | 0.79 | 0.96 |
| Fruit & berry fibre (quartiles) | | | | | | |
| g day$^{-1}$ < 1.54 | 71 (65%) | 38 (35%) | 109 | 1.0 (*n* = 439) | 1.0 (*n* = 431) | 1.0 (*n* = 428) |
| 1.54 ≤ 2.43 g day$^{-1}$ | 88 (78%) | 25 (22%) | 113 | 0.5 (0.3–1.0) | 0.6 (0.2–1.6) | 0.6 (0.3–1.1) |
| 2.43 ≤ 4.76 g day$^{-1}$ | 80 (73%) | 29 (27%) | 109 | 0.7 (0.4–1.2) | 1.4 (0.5–3.8) | 0.9 (0.5–1.7) |
| g day$^{-1}$ ≥ 4.76 | 85 (79%) | 23 (21%) | 108 | 0.5 (0.3–0.9) | 1.3 (0.5–3.5) | 0.9 (0.4–1.7) |
| Missing | 8 | 1 | 9 | | | |
| *P* for trend | | | | 0.06 | 0.41 | 0.92 |
| Vegetable fibre (quartiles) | | | | | | |
| g day$^{-1}$ < 0.678 | 71 (65%) | 38 (35%) | 109 | 1.0 (*n* = 436) | 1.0 (*n* = 429) | 1.0 (*n* = 425) |
| 0.678 ≤ 1.139 g day$^{-1}$ | 79 (72%) | 30 (28%) | 109 | 0.8 (0.4–1.3) | 0.5 (0.2–1.3) | 0.8 (0.4–1.4) |
| 1.139 ≤ 1.90 g day$^{-1}$ | 86 (79%) | 23 (21%) | 109 | 0.5 (0.3–0.9) | 0.5 (0.2–1.4) | 0.5 (0.3–1.1) |
| g day$^{-1}$ ≥ 1.90 | 86 (79%) | 23 (21%) | 109 | 0.5 (0.3–0.9) | 1.0 (0.4–2.6) | 0.6 (0.3–1.2) |
| Missing | 10 | 2 | 12 | | | |
| *P* for trend | | | | 0.01 | 0.85 | 0.09 |
| Vitamin C (quartiles) | | | | | | |
| mg day$^{-1}$ < 50.7 | 73 (72%) | 28 (28%) | 101 | 1.0 (*n* = 404) | 1.0 (*n* = 398) | 1.0 (*n* = 494) |
| 50.7 ≤ 79.1 mg day$^{-1}$ | 79 (78%) | 22 (22%) | 101 | 0.7 (0.4–1.4) | 0.1 (0.1–0.5) | 0.8 (0.4–1.6) |
| 79.1 ≤ 110.2 mg day$^{-1}$ | 72 (71%) | 29 (29%) | 101 | 1.1 (0.6–1.9) | 0.7 (0.2–2.2) | 1.1 (0.6–2.3) |
| mg day$^{-1}$ ≥ 110.2 | 76 (75%) | 25 (25%) | 101 | 0.9 (0.5–1.6) | 0.6 (0.2–1.7) | 1.1 (0.6–2.2) |
| Missing | 32 | 12 | 44 | | | |
| *P* for trend | | | | 0.92 | 0.91 | 0.58 |
| Folate (quartiles) | | | | | | |
| µg day$^{-1}$ < 139 | 55 (69%) | 25 (31%) | 80 | 1.0 (*n* = 322) | 1.0 (*n* = 317) | 1.0 (*n* = 312) |
| 139 ≤$^{1}$ 173.6 µg day$^{-}$ | 63 (78%) | 18 (22%) | 81 | 0.6 (0.3–1.3) | 0.3 (0.1–1.2) | 0.6 (0.3–1.4) |
| 173.6 ≤ 214 µg day$^{-1}$ | 57 (70%) | 24 (30%) | 81 | 0.9 (0.5–1.8) | 0.5 (0.1–1.5) | 0.7 (0.3–1.6) |
| µg day$^{-1}$ ≥ 214 | 61 (76%) | 19 (24%) | 80 | 0.7 (0.3–1.4) | 1.4 (0.4–4.8) | 1.0 (0.4–2.1) |
| Missing | 90 | 36 | 126 | | | |
| *P* for trend | | | | 0.50 | 0.62 | 0.95 |

[a]Adjusted odds ratios for HPV DNA, smoking and age. [b]Adjusted odds ratios for HPV seropositivity, smoking and age.

 *© 2000 Cancer Research Campaign*

**Table 2** Sexual history and their associations with the risk of CIN 2–3

| | Normal | CIN 2–3 | Total number | OR | Adj OR[a] | Adj OR[b] |
|---|---|---|---|---|---|---|
| Age of sexual debut | | | | | | |
| > 20 | 45 (90%) | 5 (10%) | 50 | 1.0 (*n* = 478) | 1.0 (*n* = 455) | 1.0 (*n* = 455) |
| 15–20 | 284 (72%) | 113 (28%) | 397 | 3.6 (1.4–9.2) | 5.3 (0.8–37.1) | 3.2 (0.9–10.8) |
| < 15 | 18 (58%) | 13 (42%) | 31 | 6.5 (2.0–20.9) | 2.9 (0.3–26.5) | 3.1 (0.7–13.4) |
| Never | 2 | 0 | 2 | | | |
| Missing | 0 | 0 | 0 | | | |
| *P* for trend | | | | 0.001 | 0.79 | 0.19 |
| No. of lifetime sexual partners[c] | | | | | | |
| 0–1 | 61 (92%) | 5 (8%) | 66 | 1.0 (*n* = 479) | 1.0 (*n* = 456) | 1.0 (*n* = 456) |
| 2–5 | 165 (70%) | 71 (30%) | 236 | 5.2 (2.0–13.6) | 2.2 (0.6–8.1) | 2.7 (1.0–7.3) |
| > 5 | 123 (70%) | 54 (30%) | 177 | 5.4 (2.0–14.1) | 1.1 (0.3–4.3) | 1.9 (0.7–5.3) |
| Missing | 0 | 1 | 1 | | | |
| *P* for trend | | | | 0.004 | 0.34 | 0.96 |
| Use of OC | | | | | | |
| Never | 61 (84%) | 12 (16%) | 73 | 1.0 (*n* = 480) | 1.0 (*n* = 457) | 1.0 (*n* = 457) |
| < 1 year | 58 (76%) | 18 (24%) | 76 | 1.5 (0.7–3.5) | 1.2 (0.3–4.8) | 1.5 (0.6–3.9) |
| 1–5 years | 124 (76%) | 40 (24%) | 164 | 1.6 (0.8–3.3) | 1.1 (0.3–3.5) | 1.4 (0.6–3.3) |
| > 5 years | 106 (64%) | 61 (36%) | 167 | 2.9 (1.5–5.9) | 1.6 (0.5–5.3) | 2.1 (0.9–4.9) |
| Missing | 0 | 0 | 0 | | | |
| *P* for trend | | | | 0.02 | 0.50 | 0.16 |
| Pregnancy | | | | | | |
| Never | 84 (75%) | 28 (25%) | 112 | 1.0 (*n* = 469) | 1.0 (*n* = 446) | 1.0 (*n* = 447) |
| Ever | 259 (72%) | 98 (28%) | 357 | 1.1 (0.7–1.8) | 4.5 (1.6–12.8) | 2.5 (1.3–4.9) |
| Missing | 6 | 5 | 11 | | | |

[a]Adjusted odds ratios for HPV DNA, smoking and age. [b]Adjusted odds ratios for HPV seropositivity, smoking and age. [c]The data on number of lifetime sexual partners has also been published in Kjellberg et al (1999).

**Table 3** Association between three measures of smoking and CIN 2–3 in individuals positive for HPV DNA and HPV Capsid antibodies respectively

| | Normal | CIN 2–3 | Total number | OR | Adj OR[a] | Adj OR[b] |
|---|---|---|---|---|---|---|
| Never-smoker & party-smoker | 215 (82%) | 47 (18%) | 262 | 1.0 (*n* = 468) | 1.0 (*n* = 461) | 1.0 (*n* = 459) |
| Past-smoker & smoker | 131 (64%) | 75 (36%) | 206 | 2.6 (1.7–4.0) | 2.5 (1.3–4.9) | 3.0 (1.9–4.7) |
| Missing | 0 | 0 | 0 | | | |
| Never-smoker & party-smoker | 215 (82%) | 47 (18%) | 262 | 1.0 (*n* = 468) | 1.0 (*n* = 461) | 1.0 (*n* = 459) |
| Past-smoker | 56 (68%) | 26 (32%) | 82 | 2.1 (1.2–3.7) | 2.3 (1.0–5.6) | 2.8 (1.5–5.2) |
| Smoker | 75 (61%) | 49 (39%) | 124 | 3.0 (1.8–4.8) | 2.6 (1.2–5.6) | 3.1 (1.8–5.2) |
| Missing | 0 | 0 | 0 | | | |
| Years of smoking | | | | | | |
| < 1 year | 215 (82%) | 47 (18%) | 262 | 1.0 (*n* = 464) | 1.0 (*n* = 457) | 1.0 (*n* = 455) |
| 1–9 years | 16 (64%) | 9 (36%) | 25 | 2.6 (1.1–6.2) | 3.5 (0.9–13.8) | 3.1 (1.2–7.8) |
| ≥ 10 years | 114 (64%) | 63 (36%) | 177 | 2.5 (1.6–3.9) | 2.3 (1.2–4.6) | 2.8 (1.7–4.6) |
| Missing | 1 | 3 | 4 | | | |
| Number of cigarettes/day | | | | | | |
| Never-smoker & party-smoker | 215 (82%) | 47 (18%) | 262 | 1.0 (*n* = 468) | 1.0 (*n* = 461) | 1.0 (*n* = 459) |
| Past-smoker | 56 (68%) | 26 (32%) | 82 | 2.1 (1.2–3.7) | 2.3 (1.0–5.9) | 2.8 (1.5–5.1) |
| Smokers 1–4 | 14 (74%) | 5 (26%) | 19 | 1.6 (0.6–4.8) | 0.5 (0.1–1.9) | 1.9 (0.6–6.0) |
| 5–14 | 46 (67%) | 23 (33%) | 69 | 2.3 (1.3–4.1) | 3.2 (1.2–8.4) | 2.4 (1.3–4.6) |
| ≥ 15 | 15 (42%) | 21 (58%) | 36 | 6.4 (3.1–13.3) | 5.8 (1.7–19.4) | 6.0 (2.7–13.3) |
| Missing | 0 | 0 | 0 | | | |
| P for trend | | | | < 0.001 | < 0.001 | < 0.001 |

[a]Adjusted odds ratios for HPV-DNA and age. [b]Adjusted odds ratios for HPV-Capsid and age.

A significant risk for CIN 2–3 was found for an early age at first coitus and for the number of lifetime sexual partners, but after adjusting for HPV, smoking and age this association disappeared (Table 2). Pregnancy was not a risk factor in the crude analysis, but having been pregnant was revealed as a strong risk factor in the adjusted analysis (Table 2).

Smoking history was associated with a risk for CIN 2–3 and adjusting for HPV did not affect the point estimate. Stratified analysis of the effect of covarying risk factors among HPV-positive women only has been proposed by Schiffman et al, (1993) as a preferred analysis method instead of adjusting for HPV. Such analysis was, however, not meaningful because of too few women

**Table 4a** Joint effect of smoking and HPV seropositivity

| | Normal | CIN 2–3 | OR | Adj. OR[a] | Adj. OR[b] |
|---|---|---|---|---|---|
| HPV seronegative | | | | | |
| Never-smoker | 134 (92%) | 11 (8%) | 1.0 (*n* = 459) | 1.0 (*n* = 459) | 1.0 (*n* = 452) |
| Ever-smoker | 77 (73%) | 29 (27%) | 4.6 (2.2–9.7) | 5.6 (2.6–12.0) | 5.2 (1.8–15.2) |
| HPV seropositive | | | | | |
| Never-smoker | 79 (70%) | 34 (30%) | 5.2 (2.5–10.9) | 5.2 (2.5–11.0) | 4.6 (1.6–12.9) |
| Ever-smoker | 53 (56%) | 42 (44%) | 9.7 (4.6–20.2) | 10.5 (5.0–22.4) | 7.2 (2.5–20.6) |
| Missing | 3 | 6 | | | |

[a]Adjusted odds ratios for age. [b]Adjusted odds ratios for HPV DNA and age.

**Table 4b** Joint effect of smoking and HPV DNA positivity

| | Normal | CIN 2–3 | OR | Adj. OR[a] | Adj. OR[b] |
|---|---|---|---|---|---|
| HPV DNA negative | | | | | |
| Never-smoker | 199 (98%) | 5 (2%) | 1.0 (*n* = 461) | 1.0 (*n* = 461) | 1.0 (*n* = 452) |
| Ever-smoker | 116 (91%) | 12 (9%) | 4.1 (1.4–12.0) | 4.3 (1.5–12.7) | 3.8 (1.3–11.2) |
| HPV DNA positive | | | | | |
| Never-smoker | 14 (26%) | 40 (74%) | 114 (39–333) | 110 (37–326) | 93 (31–280) |
| Ever-smoker | 13 (17%) | 62 (83%) | 190 (65–553) | 189 (65–552) | 186 (62–556) |
| Missing | 4 | 3 | | | |

[a]Adjusted odds ratios for age. [b]Adjusted odds ratios for HPV seropositivity and age.

in the reference category. There was a dose–effect relationship with the number of cigarettes smoked per day (Table 3). Possible interaction between HPV16 and smoking was explored using the jointly negative as the reference category (Olsen et al, 1998). Smoking increased the point estimate of the CIN 2–3 risk both among HPV DNA positive and negative women and both among HPV seropositive and seronegative women, but there was no evidence of interaction (Table 4).

### DISCUSSION

This is one of only few population-based studies of the CIN 2–3 risk associated with major environmental risk factors such as smoking, diet and oral contraceptives that has taken HPV exposure into account. Earlier studies have commonly been contradictory. Micronutrients such as folic acid, vitamins A, E, C and β-carotene have in some studies shown a protective effect on cervical cancer and CIN (Cuzick et al, 1990; Slattery et al, 1990; Herrero et al, 1991; Liu et al, 1993) and some other studies not (Ziegler et al, 1991; Palan et al, 1998; Wideroff et al, 1998). In our study, we could not show any protective effect of these dietary components and risk for CIN 2–3, not even before adjustment for HPV. We measured food intake and not serum levels of micronutrients, but neither this study nor some of the studies that also analysed serum levels of micro-nutrients could show any association to CIN 2–3 (Butterworth et al, 1992; Ho et al, 1998). Oral contraceptive pill intake has been postulated to induce a local folic acid deficiency and interfere with DNA synthesis, repair or somehow alter the susceptibility of cells to oncogenic viruses or chemical carcinogens and therefore be a risk factor for CIN (Butterworth et al, 1982; Harper et al, 1994). We found no association of folate intake with CIN 2–3 in the first place and also did not detect any interaction between folate intake and OC use. The risk associated with prolonged OC use was entirely explained by taking HPV into account.

The dietary associations found in this study (increased risk associated with boiled coffee and with high intake of dairy products) have not been consistently found in previous studies and the possibility exists that they may be chance associations attributable to multiple hypothesis testing. Further studies would be needed to evaluate the consistency of these risks. As earlier shown, retrospective collection of dietary information can lead to misclassification and to biased estimates as compared to prospectively collected dietary information, i.e. ‘recall bias’ (Michaelsson et al, 1996).

In the present study, cofactors were studied taking both HPV DNA presence (present/persistent infection) and HPV seropositivity (cumulative exposure) into account. This is intended to allow distinguishing between cofactors independent of HPV (not affected by HPV adjustments), cofactors confounded by risk of HPV exposure (eliminated both by adjusting for HPV seropositivity and for HPV DNA) and cofactors that determine whether HPV is cleared or becomes persistent (eliminated by adjusting for HPV DNA, not affected by adjusting for HPV seropositivity). However, HPV seropositivity is not completely sensitive (estimated at 50–70% sensitivity; Kjellberg et al, 1999) and attenuation but not elimination of risks after adjusting for HPV seropositivity may also reflect residual confounding.

Sexual history is an established risk factor for CIN and cervical cancer. In a previous paper, we have found that HPV-infection explained this association and that sexual history per se thus was not a risk factor (Kjellberg et al, 1999). In this study we detected the well known association between early age of sexual debut and risk for CIN, but again this association lost significance after adjusting for HPV infection.

Parity has repeatedly been found to be a cervical cancer risk factor (Schiffmann and Brinton, 1995) also after taking HPV DNA into account (Schiffmann et al, 1993). Causal models that have been proposed are trauma to the cervix or hormonal effects

*© 2000 Cancer Research Campaign*

(Schiffman and Brinton, 1995). Since very few women in our study were multiparous, we investigated a possible effect of pregnancy. There was no association in the crude analyses, but a strong association was revealed after taking HPV into account. Our study thus suggests that pregnancy may both increase the risk for CIN 2–3 and protect against HPV infection. Previous literature on the interaction between HPV infection and pregnancy has not been consistent and further studies on the subject would seem to be warranted.

The effects of smoking have been well studied and show a strong association to CIN and cervical cancer (Cuzick et al, 1990; Winkelstein, 1990; Phillips and Smith, 1994; Ho et al, 1998; Kruger-Kjaer et al, 1998; Olsen et al, 1998). Chemical carcinogens in tobacco, such as cotinine and nicotine, probably exert a mitogenic effect by activating carcinogenic nitrosamines and causing DNA damage and also may impair the local immune defence in the cervical epithelium. However, the fact that smoking as a behaviour in some populations is associated with a lifestyle with increased risk for HPV infection has suggested that the association seen with smoking may be due to residual confounding from HPV or other sexually transmitted pathogens (Winkelstein, 1990; Ho et al, 1998). The fact that the significant excess risk for CIN 2–3 associated with smoking was not reduced by adjustment for HPV indicates that smoking does indeed have a causal association with CIN 2–3. This is also supported by reports indicating that smoking cessation facilitates regression of CIN (Szarewski et al, 1996). Whether smoking is an entirely independent risk factor or works as a risk modifier of HPV exposure could not be disclosed in the present study.

In summary, we found little evidence that variations within the normal Swedish food intake influences the risk for cervical neoplasia, nor that oral contraceptive use per se influences this risk. By contrast, the association with smoking was dose-dependent and could not be explained by confounding by HPV. The present study supports the concept that the risk associated with smoking is causal.

### ACKNOWLEDGEMENTS

We thank Sören Holmgren, Department of Nutritional Research and Pathology for managing the database. Supported by grants from Lion's Research Foundation, Umeå (AMP 97-116) and by the Swedish Cancer Society.

### REFERENCES

Geijersstam V, Kibur M, Wang Z, Koskela P, Pukkala E, Schiller JT, Lehtinen M and Dillner J (1998) Stability over time of serum antibody levels to human papillomavirus type 16. *J Infect Dis* **177**: 1710–1714

Andersson-Ellström A, Dillner J, Hagmar B, Schiller J, Sapp M, Forssman L and Milsom I (1996) Comparison of development of serum antibodies to HPV16 and HPV33 and aquisition of cervical HPV DNA among sexually experienced and virginal young girls. A longitudinal cohort study. *Sexually Transm Dis* **23**: 234–238

Bauer HM, Ting Y, Greer CE, Chambers JC, Tashiro CJ, Chimera J, Reingold A and Manos MM (1991) Genital human papillomavirus infection in female university students as determined by a PCR-based method. *JAMA* **265**: 472–478

Burger MPM, Hollema H, Gouw ASH, Pieters WJLM and Quint WGV (1993) Cigarette smoking and human papillomavirus in patents with reported cervical cytological abnormality. *Br Med J* **306**: 749–752

Butterworth CE, Hatch KD, Gore H, Mueller H and Krumdieck CL (1982) Improvement in cervical dysplasia associated with folic acid therapy in users of oral contraceptives. *Am J Clin Nutr* **35**: 73–82.

Butterworth CE, Hatch KD, Macaluso M, Cole P, Sauberlich HE, Soong S-J, Borst M and Baker VV (1992) Folate deficiency and cervical dysplasia. *JAMA* **267**: 528–533

Cuzick J, De-Stavola BL, Russel MJ and Thomas BS (1990*a*) Vitamin A, vitamin E and the risk of cervical intraepithelial neoplasia. *Br J Cancer* **62**: 651–652

Cuzick J, Singer A, De-Stavola BL and Chomet J (1990*b*) Case-control study of risk factors for cervical intraepithelial neoplasia in young women. *Eur J Cancer* **26**: 684–690

Cuzick J, Terry G, Ho L, Hollingworth T and Anderson M (1994) Type-specific human papillomavirus DNA in abnormal smears as a predictor of high-grade cervical intraepithelial neoplasia. *Br J Cancer* **69**: 167–171

Dillner J, Lehtinen M, Börge T, Luostarinen T, Youngman L, Jellum E, Koskela P, Gislefoss RE, Hallmans G, Paavonen J, Sapp M, Schiller JT, Hakulinen T, Thoresen S and Hakama M (1997) Prospective seroepidemiological study of human papillomavirus infection as risk factor for invasive cervical cancer. *J Natl Cancer Inst* **89**: 1293–1299

Eluf-Neto J, Booth M, Munoz N, Bosch FX, Meijer CJLM and Walboomers JMM (1994) Human papillomavirus and invasive cervical cancer in Brazil. *Br J Cancer* **69**: 114–119

Evander M, Edlund K, Boden E, Gustavsson Å, Jonsson M, Karlsson R, Rylander E and Wadell G (1992) Comparison of a one-step and a two-step polymerase chain reaction with degenerate general primers in a population-based study of human papillomavirus infection in young Swedish women. *J Clin Microbiol* **30**: 987–992

Harper JM, Levine AJ, Rosenthal DL, Wiesmeier E, Hunt IF, Swendseid ME and Haile RW (1994) Erythrocyte folate levels, oral contraceptive use and abnormal cervical cytology. *Acta Cytol* **38**: 324–330

Herrero R, Potischman N, Brinton LA, Reeves WC, Brenes MM, Tenorio F, de Britton RC and Gaitan E (1991) A case–control study of nutrient status and invasive cervical cancer. I. Dietary indicators. *Am J Epidemiol* **134**: 1335–1346

Hildesheim A, Reeves WC, Brinton LA, Lavery C, Brenes M, De-La Guardia ME, Godoy J and Rawls WE (1990) Association of oral contraceptive use and human papillomaviruses in invasive cervical cancers. *Int J Cancer* **45**: 860–864

Ho GYF, Kadish RDB, Basu J, Palan PR, Mikhail M and Romney SL (1998*a*) HPV 16 and cigarette smoking as risk factors for high-grade cervical intra-epithelial neoplasia. *Int J Cancer* **78**: 281–285

Ho GYF, Palan PR, Basu J, Romney SL, Kadish AS, Mikhail M, Wasserteiler-Smoller S, Runowicz C and Burk RD (1998*b*) Viral characteristics of human papillomavirus infection and antioxidant levels as risk factors for cervical dysplasia. *Int J Cancer* **78**: 594–599

Ho GYF, Bierman R, Beardsley L, Chang CJ and Burk RD (1998*c*) Natural history of cervicovaginal papillomavirus infection in young women. *N Engl J Med* **338**: 423–428

Johansson I, Hallmans G, Eriksson S, Hagman U, Bruce Å, Wikman Å, Kaaks R and Riboli E (1994) Evaluation of the accuracy of a dietary questionnaire aimed for the Västerbotten study. *Scand J Nutr* **38**: 50–55

Kjellberg L, Wiklund F, Sjöberg I, Wadell G, Ångström T, Dillner J and Mählck CG (1998) A population-based study of HPV DNA testing for predicting cervical intraepithelial neoplasia. *Am J Obstet Gynecol* **179**: 1497–1502

Kjellberg L, Wang Z, Wiklund F, Edlund K, Ångström T, Lenner, P, Sjöberg I, Hallmans G, Wallin K-L, Sapp M, Schiller J, Wadell G, Mählck C-G and Dillner J (1999) Sexual Behavior and papillomavirus exposure in cervical intraepithelial neoplasia: a population based case–control study. *Journal of General Virology* **80**: 391–398

Kruger-Kjaer S, van den Brule AJC, Svare EI, Engholm G, Sherman ME, Poll PA, Walboombers JMM, Bock JE and Meijer CJLM (1998) Different risk factor patterns for high-grade and low-grade intraepithelial lesions on the cervix among HPV-positive and HPV-negative young women. *Int J Cancer* **76**: 613–619

Kwasniewska A, Charzewska J, Tukendorf A and Semczuk M (1998) Dietary factors in women with dysplasia colli uteri associated with human papillomavirus infection. *Nutr Cancer* **30**: 39–45

Liu T, Soong SJ, Wikson NP, Craig CB, Cole P, Macaluso M and Butterworth CE Jr (1993) A case–control study of nutritional factors and cervical dysplasia. *Cancer Epidemiol Biomakers Prevent* **2**: 525–530

Lorincz AT, Reid R, Jenson AB, Greenberg MD, Lancaster W and Kurman RJ (1992) Human papillomavirus infection of the cervix: relative risk associations of 15 common anogenital types. *Obstet Gynecol* **79**: 328–337

Michaelsson K, Holmberg L, Ljunghall S, Mallmin H, Persson P-G and Wolk A (1996) Effect of prefracture versus postfracture dietary assessment on hip fracture risk estimates. *Int J Epidemiol* **25**: 403–410

Olsen AO, Dillner J, Gjoen K and Magnus P (1997) Seropositivity against HPV 16 capsids: a better marker of past sexual behavior than presence of HPV DNA. *Genitourin Med* **73**: 131–135

*© 2000 Cancer Research Campaign*

Olsen AO, Dillner J, Skrondal A and Magnus P (1998) Combined effect of smoking and human papillomavirus type 16 infection in cervical cancerogenesis. *Epidemiology* **9**: 346–349
Östör AG (1993) Natural history of cervical intraepithelial neoplasia: a critical review. *Int J Gynecol Pathol* **12**: 186–192
Palan PR, Chang CJ, Mikhail MS, Ho GY, Basu J and Romney SL (1998) Plasma concentrations of micronutrients during a nine-month clinical trial of beta-carotene in women with precursor cervical cancer lesions. *Nutr Cancer* **30**: 46–52
Phillips AN and Smith GD (1993) Smoking and human papillomavirus infection. *BMJ* **306**: 1268–1269
Schiffman MH and Brinton LA (1995) The epidemiology of cervical carcinogenesis. *Cancer* **76**: 1888–1901
Schiffman MH, Bauer HM, Hoover RN, Glass AG, Cadell DM, Rush BB, Scott DR, Sherman ME, Kurman RJ, Wacholder S, Stanton CK and Manos MM (1993) Epidemiologic evidence showing that human papillomavirus infection causes most cervical intraepithelial neoplasia. *J Natl Cancer Inst* **85**: 958–964
Slattery ML, Abbott TM, Overall JC, Robinson LM, French TK, Jolles C, Gardner JW and West DW (1990) Dietary vitamins A, C, and E and selenium as risk factors for cervical cancer. *Epidemiology* **1**: 8–15
Szarewski A, Jarvis MJ, Sasienin P, Anderson M, Edwards R, Steele SJ, Guillebaud J and Cuzick J (1996) Effect of smoking cessation on cervical lesion size. *Lancet* **347**: 941–943
Wideroff L, Potischman N, Glass AG, Greer CE, Manos MM, Scott DR, Burk RD, Sherman ME, Wacholder S and Schiffman M (1998) A nested case-control study of dietary factors and the risk of incident cytological abnormalities of the cervix. *Nutr Cancer* **30**: 130–136
Winkelstein W (1990) Smoking and cervical cancer – current status: a review. *Am J Epidemiol* **131**: 945–958
Ziegler RG, Jones CJ, Brinton LA, Norman SA, Mallin K, Levine RS, Lehman HF, Hamman RF, Trumble AC and Rosental JF (1991) Diet and the risk of in situ cervical cancer among white women in the United States. *Cancer Causes Control* **2**: 17–29

*© 2000 Cancer Research Campaign*
